# Cases Read before the Chicago Medical Society

**Published:** 1864-01

**Authors:** Hiram Wanzer

**Affiliations:** Chicago, Ill.


### ARTICLE IV.

### CASES READ BEFORE THE CHICAGO MEDICAL SOCIETY. FRIDAY EVENING, NOV. 13, 1863.

BY HIRAM WANZER, M.D., of Chicago, Ill.

MR. PRESIDENT AND GENTLEMEN.—I present you two pathological specimens. One a cancer of the uterus; the other a bony specimen of comminuted fracture. I have lately had three interesting cases of injury of the knee-joint, which I will relate; together with other cases which may be of interest to the Society.

The first case was a compound comminuted fracture of the tibia and fibula at the upper third, caused by a rail-car passing over the limb, crushing the bones in numerous fragments, and reducing the neighboring soft parts to a mere pulp. Stimulants were freely given to promote reaction, Dr. FISHER was sent for. By the time we were ready for operating, reaction, we thought, was sufficiently established. I amputated at the lower third of the thigh. Only the femoral artery required ligation. This was about 5 o'clock P.M. I requested the family to send for me at 4 o'clock the following morning, should he be living.

As the messenger was leaving the room, the patient rallied and conversed with his friends. Not 15 minutes elapsed, and before the messenger had time to reach my office, he sank in the arms of death, without a struggle.

To me there appears something singular in this sudden death. He had been semi-comatose up to 15 minutes before death, when he suddenly rallied, cheering friends with bright prospects of a certain recovery. Many of the Society, older in the profession than myself, having witnessed many death-bed scenes, can perhaps better account for it.

The second case, a German, æt. 30, was run over by a rail-car, receiving a compound comminuted fracture of the tibia at its upper third; also fracturing one of the condyles of the femur.

The bony specimen is before you. The soft parts were in the same condition as the case previously mentioned. I dispatched a messenger for Prof. E. ANDREWS, who, upon his arrival, examined the injury and found that the fracture extended to the cavity of the knee-joint. He advised, as the only alternative, for the patient, immediate amputation. I amputated at the lower third of the thigh, as near the joint as possible. Only the femoral artery and a small muscular branch required ligation. The patient had long made too liberal use of the various alcoholic drinks. The fourth night after amputation, delirium traumaticum supervened, and it became necessary to give freely his accustomed beverage, which overcame these symptoms. But while we were overcoming the delirium on the one hand by the stimuli, we were at the same time inducing a hyper-excitement of the heart and arteries on the other. In a very short period, alarming secondary hemorrhage from the femoral artery occurred, which, however, was speedily controlled by compression over Poupart's ligament. The hemorrhage recurred at intervals for one week; so that it became necessary to withdraw stimulants altogether. Will that blissful period ever dawn upon our profession, when some substitute for alcohols, in medicine, will be devised. They carry with them a polution and a degradation which can never be washed away; and our profession to-day are in part responsible for their inordinate use. But this was not the only pathological feature in the case. Traumatic erysipelas occurred in the stump, resulting in suppuration and sloughing of a part of the soft parts of the thigh. A free incision obviated a part of this difficulty.

The stump now looks well, and is healing rapidly by granulation; and I regard the patient as completely out of danger.

The third case was an injury of the soft parts in the popliteal space, without fracture. The man fell upon the railroad track. The wheels of a loaded wagon passed over the limb, dividing the soft parts transversely, nearly across the popliteal space, just below the joint. The internal head of the gastrocnemi muscle was divided, together with the internal articular arteries, veins, and nerves; and I apprehend the popliteal artery also. The instantaneous hemorrhage was alarming. When I arrived he was apparently exsanguine, and exhausted from loss of blood. The shock I consider greater in this case than either of those we amputated. As the hemorrhage had ceased before I arrived, I flexed the leg upon the thigh, it being the only way the parts could be brought in apposition. This method assisted also in preventing future hemorrhage. Several interrupted sutures, compress, and strong bandage were all the treatment necessary at this time. Reaction was not established to any degree until the next morning.

I continued flexion until the wound was nearly healed and the danger of secondary hemorrhage passed. The same symptoms of delirium traumaticum supervened, as with the other, and it became necessary to give him freely his accustomed beverage, until this danger was overcome; supperadded to these aggravated symptoms, there was a phagadenic inflammation of the superficial structures of the thigh. The granulations assumed a most unhealthy appearance. The suppuration extended along the fascia and flexor muscles of the thigh to near the hip. This suppurative diathesis was soon overcome by generous diet, large and frequent doses of tinct. ferri muriatis. The matter was localized by a compress and strong bandage above. I commenced extension before the wound entirely united. At present there is no perceptible shortening, and he walks easily with a cane.

The fourth case was a dislocation of the shoulder. The head of the humerus was thrown forward under the clavicle, and felt there distinctly. There was undue prominence of the acromion process. I placed him in the recumbent posture, and with my heel in the axilla made extension in the manner I have previously, where the head of the humerus was dislocated downwards in the axilla. This manipulation proved unavailing. As the dislocation was sub-clavicular it was necessary to make extension obliquely downwards and backwards, it being in a direct line with the displacement. By this most simple method the humerous was easily dislodged and entered the glenoid cavity. I would ask the Society, how many dislocations of this kind they have met with? As our books do not make frequent mention of them.

The fifth and last case I will present you this evening was cancer of the uterus. I was called to Mrs. T., æt. 44, mother of five interesting children. She had been suffering with cancer of the uterus six months. The intolerable odor emitted led me to diagnose the case as cancer upon my first entrance into her chamber. A few of her more general symptoms I will briefly describe. There was severe lancinating, hypogastric, lumbar, and femoral pains, often amounting to agony. There was an occasional sanguinolent, and a constant fetid discharge from the vagina. Her countenance was hippocratic, and there was extreme emaciation, a yellow tinge of the skin, and hectic fever. Constant vesical and rectal irritation, and continual constipation. The bowels were never moved without cathartics or laxative enemas. Her appetite remained unusually good. These were the symptoms when I was first called, significant of malignant disease. A digital examination revealed the lower fifth of the recto-vaginal septum and the posterior lip of the os and cervix uteri converted into a mass of schirrus. The anterior lip of the os and cervix uteri had previously disappeared from ulceration. The schirrus mass mentioned sloughed away one week previous to her death, opening the vagina and rectum into one passage; and I apprehend the bladder became perforated also. The irritation of the intestinal and urinary discharges became then so great that I think it hastened her dissolution. She lived seven weeks after my first visit. The treatment was only paliative: large doses of morphia frequently given to allay pains; strong laxative enemas and chlorine washes constituted the major part of the treatment. Autopsy revealed what you see. The fundus and body of the uterus a mass of schirrus. The os, cervix, and lower part of the body of the uterus, and lower fifth of the recto-vaginal septum entirely sloughed away by ulceration. The ovaries, fallopian tubes, broad ligaments, and the other appendages of the uterus, do not seem to be involved in the disease.

Dr. JOHN BARTLETT assisted me at the *post mortem* Had I had time, I should have invited other members of the Society. In conclusion, I must apologize to the Society for the imperfect manner I have presented these cases.

